# Multidisciplinary management of locally recurrent rectal cancer with carbon ion radiotherapy followed by prophylactic removal of the irradiated bowel: a case report

**DOI:** 10.1186/s40792-024-01811-2

**Published:** 2024-01-10

**Authors:** K. Nagata, H. Takiyama, K. Tashiro, M. Yamadera, K. Okamoto, Y. Kajiwara, E. Shinto, Y. Kishi, S. Matsukuma, S. Yamada, H. Ueno

**Affiliations:** 1 Department of Surgery, National Defense Medical College, 3-2 Namiki, Tokorozawa, Saitama 359-8513 Japan; 2 QST Hospital, National Institutes for Quantum Science and Technology, 4-9-1 Anagawa, Inage-ku, Chiba, 263-8555 Japan; 3 Department of Pathology and Laboratory Medicine, National Defense Medical College, 3-2 Namiki, Tokorozawa, Saitama 359-8513 Japan

**Keywords:** Locally recurrent rectal cancer, Carbon ion radiotherapy, Radiation-exposed bowel removal

## Abstract

**Background:**

Locally recurrent rectal cancer (LRRC) involving the upper sacrum is typically incurable, and palliative treatment is the only option for most patients, resulting in a poor prognosis and reduced quality of life. Carbon ion radiotherapy (CIRT) has emerged as a promising modality for treating LRRC. This report presents a case of LRRC with sacral involvement that was managed via multidisciplinary therapy incorporating CIRT.

**Case presentation:**

A 55-year-old male was diagnosed with an anastomotic recurrence of rectal cancer 15 months after undergoing anterior resection. Computed tomography (CT) suggested that the lesion was at an anastomosis site and broadly adherent to the upper sacrum, and colonoscopy confirmed the diagnosis of LRRC. Histopathological examination of the biopsy specimens revealed adenocarcinoma cells and that lesion was genetically RAS-wild. Induction chemotherapy with mFOLFOX6 and panitumumab was used as the first treatment. The recurrent lesion shrank and no signs of distant metastasis were observed after 11 cycles, although the range of the lesions attached to the sacrum remained unchanged. Therefore, we provided CIRT for this inoperable lesion and prophylactically removed the radiation-exposed bowel including the recurrent lesion, because radiation-induced ulcers can cause bleeding and perforation. Despite the presence of considerable fibrosis in the irradiated region, the operation was successful and the postoperative course had no untoward incidents. He is still recurrence-free 24 months following surgery, despite the lack of adjuvant chemotherapy. This is the first report of CIRT followed by CIRT-irradiated bowel removal for an unresectable anastomosis recurrent lesion.

**Conclusions:**

The clinical course of this case suggests that CIRT could be a potentially effective therapeutic option for LRRC involving the bowel, as long as the prophylactic removal of the irradiated bowel is performed at the optimal time. Further research involving larger sample sizes is warranted to validate the findings and conclusions of this case report.

## Background

The incidence of locally recurrent rectal cancer (LRRC) has decreased because of advances in precise preoperative magnetic resonance imaging assessment, surgical techniques, and preoperative chemoradiotherapy for primary rectal cancer [1]. Nevertheless, in Japan, the incidence of LRRC after radical surgery is 4.1% and 1.3% in the pelvic and anastomotic sites, respectively [2]. LRRC is associated with poor prognosis, reduced quality of life, and increased use of medical resources. Moreover, only a few patients are eligible for curative surgical resection [3].

Surgery remains the primary course of treatment for resectable LRRC [3]. If surgery is not possible, chemotherapy and/or radiation may be provided until a conversion procedure is performed or patient intolerance is noted [4, 5]. Current treatment modalities for LRRC warrant improvement to manage cases where localized recurrence with invasion of other organs is observed, surgical resection is technically possible but would require performing a highly invasive procedure such as sacral resection and total pelvic exenteration, or the risk of complications is high. Previous studies have shown that carbon ion radiotherapy (CIRT) is a safe and effective treatment for unresectable LRRC with excellent local control and survival benefits [6–9]. Therefore, CIRT has been recognized as a modality of curatively intended treatment with great promise for LRRC. Moreover, Japan’s Ministry of Health, Labour and Welfare authorized insurance coverage for CIRT for managing postoperative unresectable local recurrence of colorectal cancer in April 2022. CIRT has generally not been indicated for patients whose planned irradiation area includes the intestinal tract, which has a high risk of causing adverse events, such as ulcer bleeding or perforation. However, if the restrictions related to the proximity of the gastrointestinal tract could be resolved, CIRT could be provided in such cases. One potential approach would be the administration of CIRT followed by prophylactic surgical removal of the irradiated gastrointestinal tract prior to the onset of expected ulcerations. We present a case of unresectable anastomotic recurrence that was managed using a combination of systemic chemotherapy, CIRT, and prophylactic exposed bowel removal, highlighting a possible multidisciplinary treatment strategy for initially unresectable LRRC.

## Case presentation

A 55-year-old male who underwent a radical surgical procedure was referred to our medical facility for further evaluation and management of a local recurrence of anastomosis. The patient previously underwent laparoscopic high anterior resection and Japanese D2 lymph node dissection after being diagnosed with rectosigmoid cancer at the age of 53 years. According to the ninth edition of the Union for International Cancer Control [10], rectal cancer was pathologically diagnosed as pT3N0M0 Stage IIA. After surgery, the patient received adjuvant therapy of uracil–tegafur and leucovorin for a year at the previous hospital. He was symptom-free for 1 year and 3 months, and no signs of recurrence were observed.

**Fig. 1:**
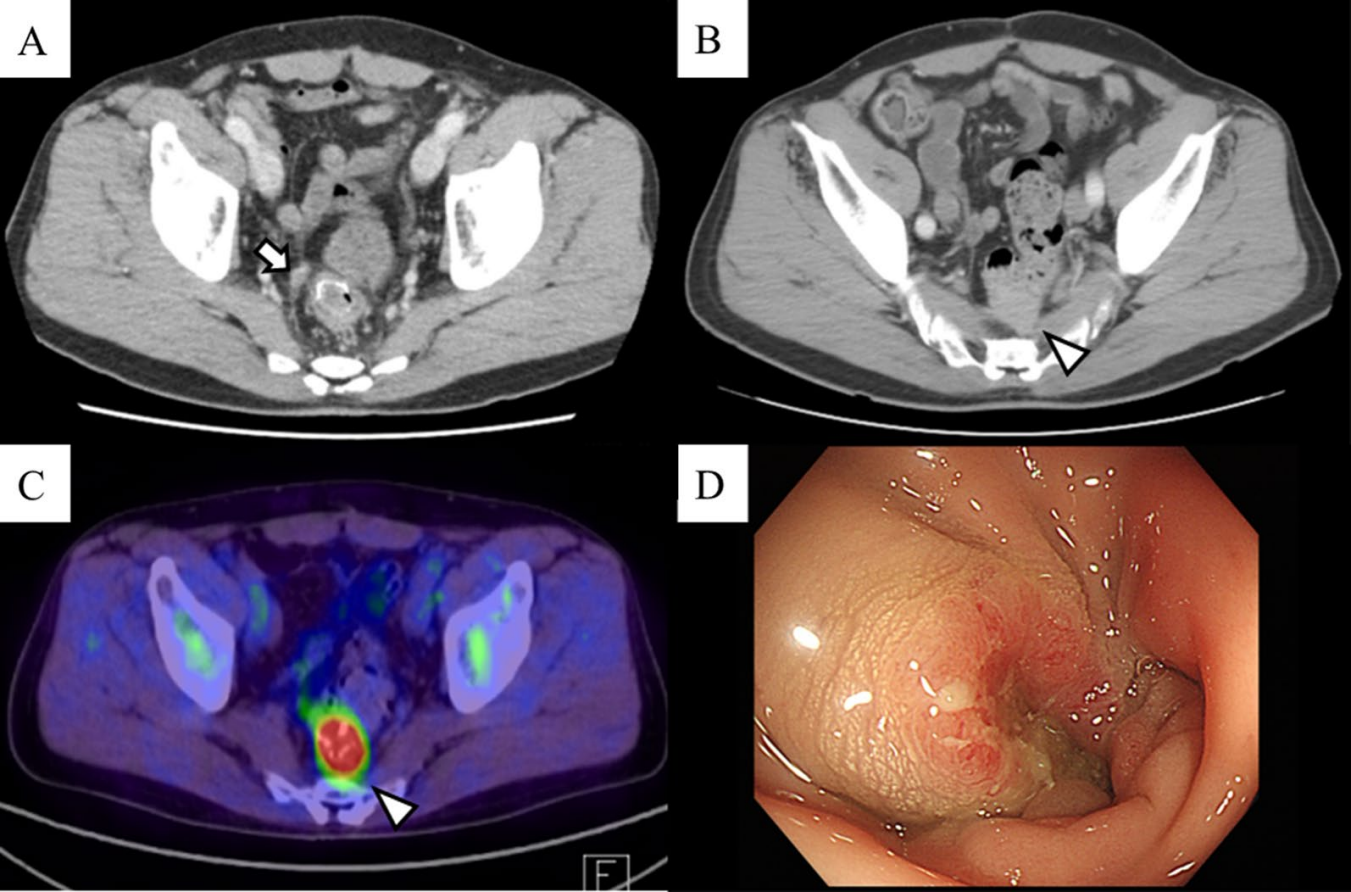
Imaging and colonoscopy findings at the time of the recurrence diagnosis. A small 10-mm nodule was detected on the ventral right side of the anastomosis (arrow) (**A**). The recurring neoplasm that emerged on the anastomosis was close to the sacrum (arrow head) (**B**). The positron emission tomography scan revealed abnormal 2-[18F]-fluoro-2-deoxy-d-glucose accumulation in the vicinity of the anastomosis (arrow head) (**C**). A recurrent lesion with an approximately half-circumference and ulceration was detected on the dorsal side of the anastomosis. Malignant neoplastic cells were also identified in the pathological examination of the biopsy specimen obtained from the anastomotic recurrent tumor (**D**)

**Fig. 2:**
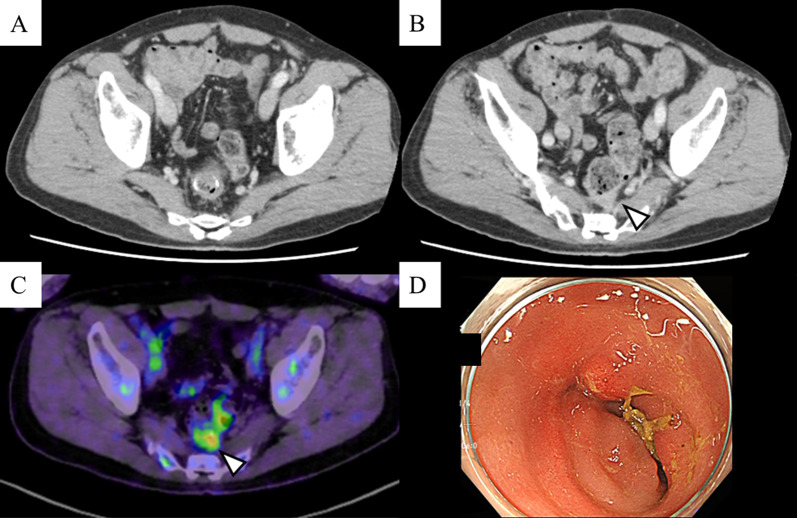
Imaging and colonoscopy findings after systemic chemotherapy. A small nodule was disappeared on the ventral right side of the anastomosis (**A**). The recurring neoplasm remained close to the sacrum (**B**). The positron emission tomography scan revealed less abnormal 2-[18F]-fluoro-2-deoxy-d-glucose accumulation in the vicinity of the anastomosis than the previous one (**C**). Colonoscopy confirmed the shrinking and flattening of the recurrent lesion (**D**)

One year and 3 months after the surgery, the serum level of carcinoembryonic antigen increased to 20.2 ng/mL. CT scan indicated the development of a tumor at the anastomosis site that was massively attached to the upper part of the sacrum and a small nodule, and it was suspected that peritoneal dissemination was present on the ventral right side of the anastomosis (Fig. 1A, B). In addition, a positron emission tomography–CT scan confirmed an abnormally high accumulation of 2-[18F]-fluoro-2-deoxy-D-glucose on the recurrent lesion (Fig. 1C). A colonoscopy confirmed the diagnosis of LRRC (Fig. 1D), and histopathological examination of the biopsy specimens revealed adenocarcinoma cells. LRRC was deemed unresectable for R0 clearance and was genetically characterized as RAS wild type. Therefore, we administered systemic chemotherapy with mFOLFOX6 and panitumumab. The recurrent tumor shrank after 11 rounds of treatment, the diminutive nodule near the anastomosis disappeared, and no signs of distant metastasis were observed. However, the recurring tumor remained in contact with the upper part of the sacrum (Fig. 2). We determined that radical surgical resection was still not possible, and CIRT facility was consulted. It was determined that CIRT could not be performed because of the close proximity of the intestinal anastomotic site. This was decided based on the fact that the dose of CIRT is higher than the dose tolerable for the intestinal tract, and its proximity to the intestinal tract can increase the risk of ulcers, bleeding, and perforation. Therefore, it was proposed that the exposed intestinal tract should be removed within 1–2 months after CIRT considering its late toxicities. The patient provided informed consent and selected multidisciplinary therapy incorporating CIRT as the treatment option.

**Fig. 3:**
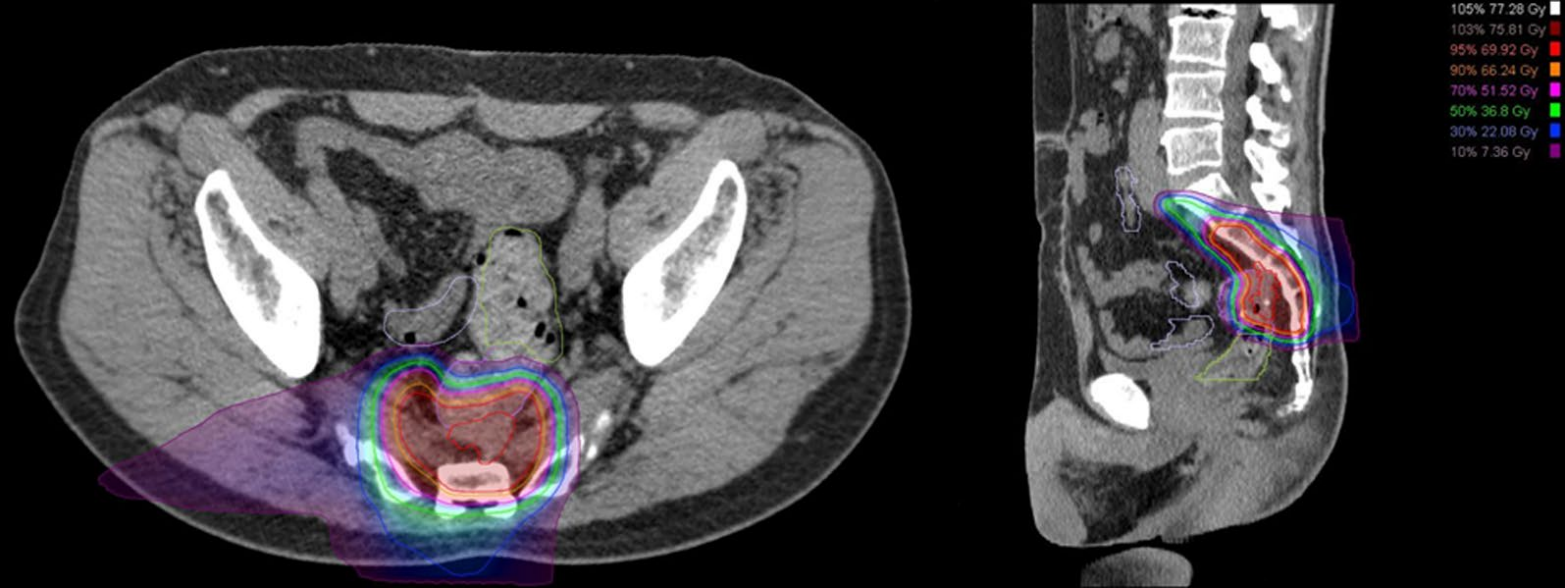
Dose distribution for carbon ion radiotherapy. The irradiation field was set up to include the recurrent lesion and the sacrum proximal to the recurrent lesion

**Fig. 4:**
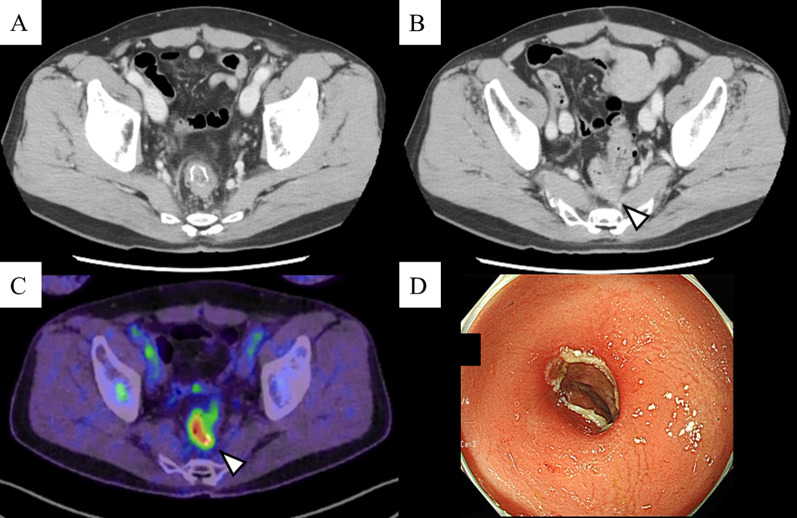
Imaging and colonoscopy findings after carbon ion radiotherapy. The diminutive nodule remained disappeared. **A** The recurrent neoplasm, which appeared on the anastomosis, decreased in size; nonetheless, there was no free space between the tumor and the sacrum (**B**). The abnormal buildup at the anastomosis was attenuated by the positron emission tomography (**C**). The anastomotic recurrent tumor was further flattened, with erythema and ulceration consistent with anastomosis site, as shown in a colonoscopy (**D**)

CIRT was administered daily 4 days/week for 16 fractions. The total irradiated dose was set at 73.6 Gy (relative biological effectiveness–weighted dose [RBE]). The radiation field included the tumor with a 10-mm margin. As a result, approximately 5 cm of the intestinal tract was included in the irradiated area (Fig. 3). The patient was re-evaluated 1 month after irradiation with multiple imaging studies, but no significant changes in the tumor were observed. In addition, there were no significant adverse effects in the intestinal tract, except for light erythema, mucosal edema, and erosion at the anastomosis (Fig. 4).

**Fig. 5:**
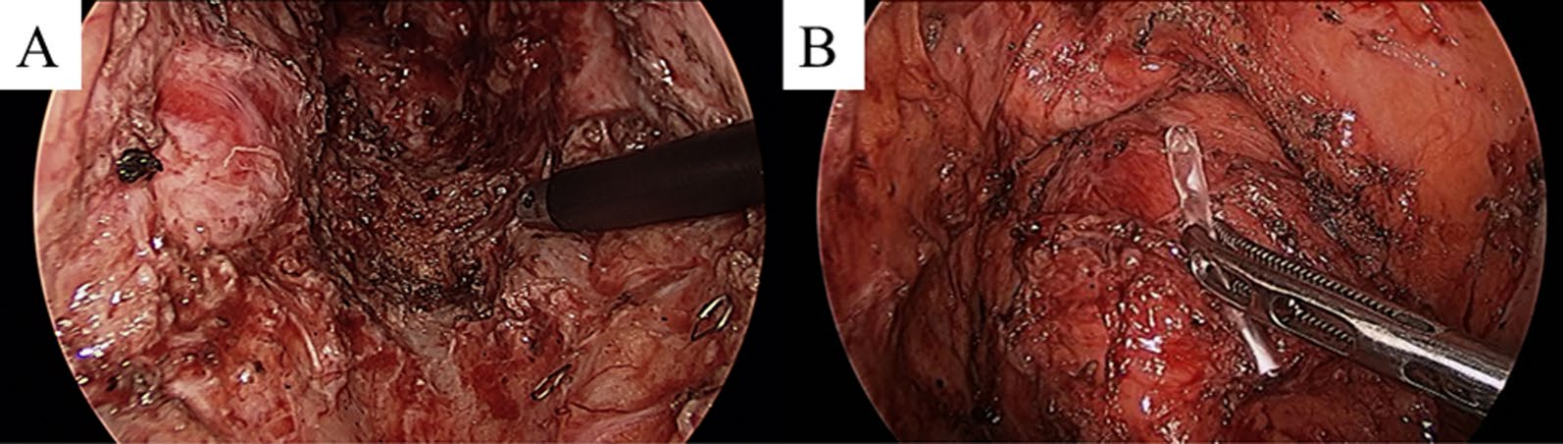
Intraoperative findings. The dorsal side of the rectal anastomosis inside the irradiated region showed considerable fibrosis (**A**). The rectal resection line was determined on 2 cm distal to the anastomosis of the previous surgery (**B**)

**Fig. 6:**
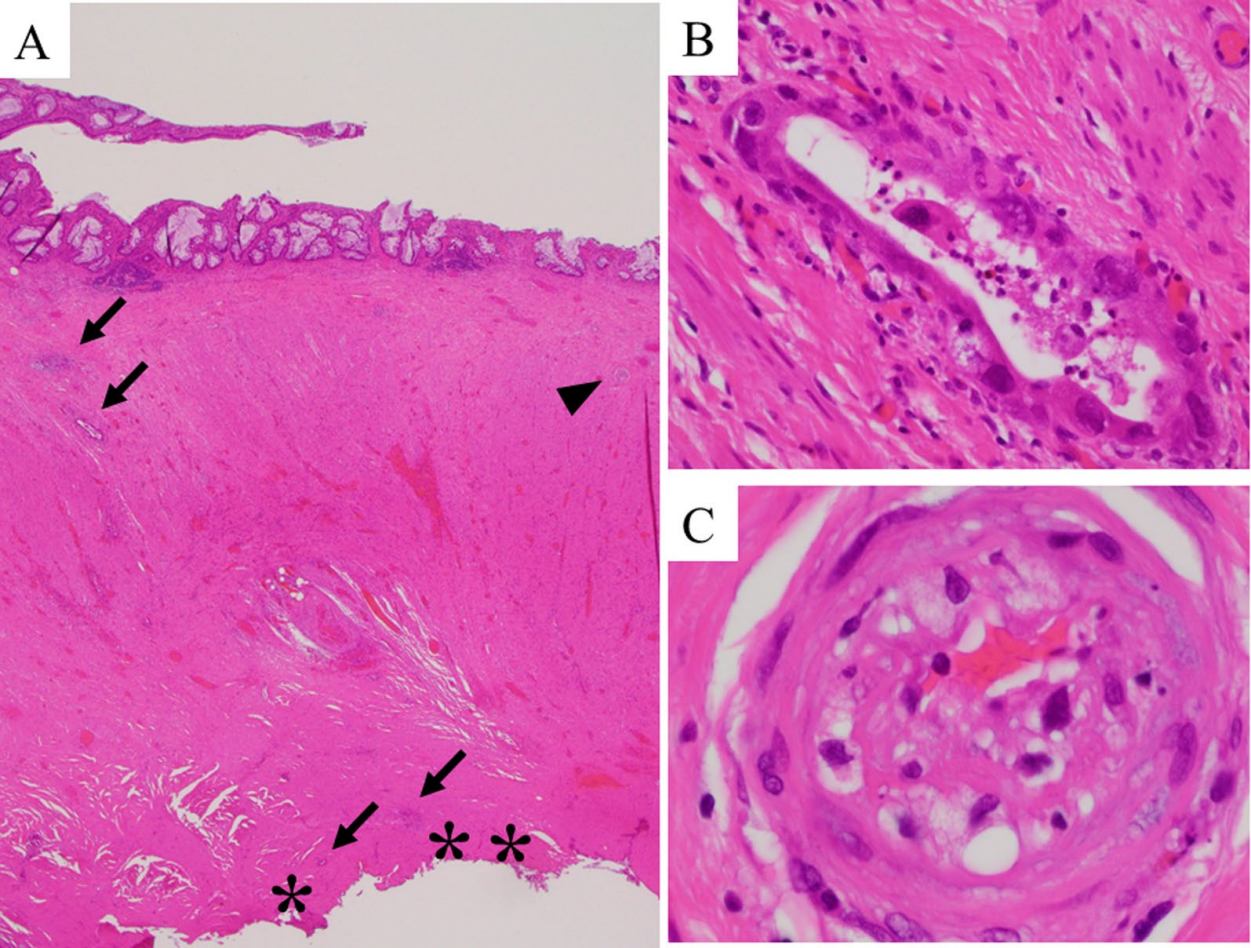
Pathological findings of surgically removed rectum. **A** Low-power views reveal massive fibrosis of the rectal wall that was exposed to the detached margin from the sacral bone (asterisks), and small numbers of scattered cancer cells (arrows) (magnification, ×12.5). **B** High-power views reveal cancer cells with markedly and irregularly swollen nuclei (magnification, ×400). **C** High-power views reveal a small vessel indicated by arrow head in Fig. 5A showing a marked luminal narrowing due to intimal thickening containing foamy cells, suggesting an irradiation effect (magnification, ×1000)

Surgical removal was performed as planned 8 weeks after CIRT. Surgery was performed laparoscopically with regional removal of the intestine. Despite the presence of adhesions that seemed to be related to the prior surgery, no significant adhesions with other organs were detected within the CIRT-irradiated area. In the irradiated area, the tissue was extremely firm owing to severe fibrosis, thus making it difficult to distinguish the dissected layer. Therefore, we proceeded with sharp dissection as close as possible to the sacrum, with the connective tissue being sharply dissected along the sacral surface using monopolar electronic scalpels and ultrasonically activated devices. This dissection line was selected to prevent any exposure of the intestinal lumen. However, sharp dissection resulted in a significant amount of exudate production, thus making it difficult to maintain a clear field of view during the surgical procedure within the pelvis. We verified the anastomosis from the prior surgery using intraoperative colonoscopy. Further, we estimated the extent of irradiated bowel based on the distance from the anastomosis using the preoperative irradiation map as a guide. The physician who designed and performed preoperative CIRT participated in the surgery and provided advice on the resection line. The rectum was resected 2 cm distal to the anastomotic tumor, and the entire irradiated bowel was removed (Fig. 5). Re-anastomosis was performed using the double-stapling technique, and a temporary loop ileostomy was constructed. No complications were observed except for a paralytic ileus, which delayed the start of meals for 7 days. Histopathological examination of the removed rectum revealed massive fibrosis and scattered small numbers of cancer cells (Fig. 6A). The nuclei of these cancer cells showed irregularly marked swelling and chromatin clumping (Fig. 6B). Because of intimal thickening that contained foamy cells, small vascular lumen was narrowed near these degenerated cancer cells (Fig. 6C). These morphological changes suggested the presence of the CIRT effect, which was estimated as TRG3 according to the previously proposed criteria [11]. No adjuvant therapy was administered, and tumor markers and imaging were performed every 3 months after the surgery. The temporary ileostomy was closed 8 months after the surgical intervention. The quality of life of the patient, especially defecation function, was maintained. The patient continues to survive with no obvious signs of recurrence 24 months after surgery.

## Discussion

Based on the positive outcome of this case, it can be inferred that a multidisciplinary approach including CIRT followed by prophylactic-irradiated intestine removal could be beneficial for patients with unresectable and CIRT untreatable LRRC. If complete resection of the tumor is possible, surgery is currently considered the only potentially curative treatment option for LRRC. The overall 5-year survival rate after R0 resection is estimated to be 40–44% [3, 12–15]. Nevertheless, previous studies have suggested that only ~ 40–50% of patients diagnosed with LRRC qualify for surgery with a curative purpose. Of these patients, approximately 30–45% could undergo R0 resection, signifying that only 20–30% of all patients with LRRC can undergo a potentially curative surgical procedure [16]. Moreover, unlike primary rectal cancer, the surgical techniques and degree of resection for LRRC are more varied and dependent on tumor location, size, and organ invasion [12, 17]. In cases where the lesion is unresectable at the time of diagnosis, individuals with good performance status may receive chemoradiotherapy to shrink the lesion and prepare for radical resection [4, 17]. Otherwise, systemic chemotherapy is only seen as a palliative treatment option for those who are unable to undergo curative surgery because of technical and anatomical constraints. Thus, there is considerable room for improvement in the treatment of patients with LRRC.

Even with stereotactic body radiation therapy or intensity-modulated radiation therapy, conventional radiotherapy for pelvic recurrence of rectal cancer has poor local control rates (68–74%) at 3 years. [18–20]. Unlike X-rays, carbon ion beams have two distinct characteristics. First, carbon ion beams have a unique dosimetric property known as the Bragg peak, which is a feature of high-energy emission at the end of their range that enables highly localized irradiation in the SOBP range. Second, its high linear energy transfer capability enables the efficient breakdown of double-stranded DNA, resulting in an enhanced biological effect. These characteristics are more useful in colorectal cancer cells that are considered radioresistant.

In 2016, Yamada et al. reported the results of 156 patients with LRRC treated with CIRT at 73.6 Gy (RBE) in 16 fractions. In that study, no grade > 3 acute reactions were observed and local control, and survival rates were 88% and 59%, respectively, at 5 years [8]. A retrospective multicenter trial on CIRT for LRRC was conducted by Shinoto et al. Local control and overall survival rates were as good as those reported in a previous report, but grade 3 acute toxicity and late toxicity were observed in 3 and 12 patients, respectively. Pelvic infection was the most common toxicity, occurring in 4% of the patients [7]. The outcomes of 77 patients treated with CIRT re-irradiation at 70.4 Gy (REB) in 16 fractions for LRRC were also reported by Yamada et al. The 3-year local control and overall survival rates were 90% and 61%, respectively. However, 8 patients (10%) experienced acute grade 3 toxicities, including pelvic infections in 5 patients and late grade 3 toxicities in 16 patients (21%). Of these 16 patients, 13 had late pelvic infections [21]. Therefore, although CIRT has great local control capabilities, pelvic infectious adverse events following CIRT should be closely monitored. CIRT is not recommended due to the high risk of adverse effects, especially in cases where the recurrent tumor is attached or has invaded the bladder or intestine.

In cases where the tumor is in close proximity with the intestinal tract but does not directly invade the tumor, recent efforts have expanded the indication of CIRT by physically separating the tumor from the surrounding organs with high risk of irradiation adverse effects using spacer insertion before CIRT [22, 23]. However, this spacer cannot be used when the recurrent lesion has directly invaded the organs at risk of irradiation adverse effects, as in this case owing to the increased risk of recurrent cancer seeding and infectious complications associated with intestinal tract injury during spacer placement surgery. Therefore, there have been few cases of its use in patients undergoing surgical intervention prior to CIRT [21, 22]. In cases where spacer insertion surgery is not possible, it may be feasible to use a multidisciplinary approach involving CIRT followed by prophylactic-irradiated intestine removal, which was used in this case. As a result, more patients will be able to receive CIRT, which may help improve the overall prognosis of unresectable LRRC. It may also be possible to cure locally advanced rectal cancer in the future, even in cases where complete tumor resection is not possible, using the exceptional local control capabilities of CIRT in a preoperative setting; clinical trials in this regard are expected in the near future.

This therapeutic approach for LRRC with an inability to achieve R0 tumor resection is based on the idea that CIRT would effectively eliminate cancer cells within the irradiated area. In this case, cancer cells were accompanied by degeneration and nuclear condensation in the resected specimen. Moreover, Hayashi et al. retrospectively reviewed seven cases of unresectable sarcoma following CIRT. The patients underwent biopsy and 12 samples were obtained from the seven cases and histological examination was performed. They observed viable tumor cells in 9 of the 12 specimens, and only 2 individuals had a relapse according to radiological findings. This implies that tumor cells in post-CIRT tissues may be deformed and dying over time following irradiation and viable tumor cells found in tissues following CIRT are not the assured cause of recurrence [24]. Careful monitoring of tumor regrowth in local remnant tissue and peritoneal dissemination is required to determine the universal validity of the treatment strategy used in our case. Our experience might extend the indication of CIRT to other types of unresectable local recurrent tumors or gynecological and bone and soft tissue tumors of the pelvis through the utilization of prophylactic removal of the irradiated intestine. As a limitation, some clinical aspects remain unclear, including the optimal timing of surgical removal of the bowel after CIRT, safety and effectiveness in larger cohorts, integration of CIRT with chemotherapy, and feasibility of generalization and promotion of this treatment method to other treatment facilities.

## Conclusion

We treated a patient who experienced localized rectal cancer recurrence using a multidisciplinary approach that included CIRT followed by prophylactic bowel removal. Preoperative CIRT may be a valuable treatment option for patients with LRRC who have no prospect of achieving complete tumor resection. Further research with larger sample sizes is warranted to confirm the findings and conclusions of this case report.

## Data Availability

Not applicable.

## Abbreviations

CIRT: Carbon ion radiotherapy
CT: Computed tomography
JSCCR: Japanese Society for Cancer of the Colon and Rectum
LRRC: Locally recurrent rectal cancer
